# Modulation of Phenylalanine and Tyrosine Metabolism in HIV-1 Infected Patients with Neurocognitive Impairment: Results from a Clinical Trial

**DOI:** 10.3390/metabo10070274

**Published:** 2020-07-03

**Authors:** Giuseppe P. Innocenti, Letizia Santinelli, Luca Laghi, Cristian Borrazzo, Claudia Pinacchio, Mariangela Fratino, Luigi Celani, Eugenio N. Cavallari, Carolina Scagnolari, Federica Frasca, Guido Antonelli, Claudio M. Mastroianni, Gabriella d’Ettorre, Giancarlo Ceccarelli

**Affiliations:** 1 Department of Public Health and Infectious Diseases, Sapienza, University of Rome, viale del Policlinico 155, 00161 Rome, Italy; giuseppepietro.innocenti@uniroma1.it (G.P.I.); cristian.borrazzo@uniroma1.it (C.B.); claudia.pinacchio@uniroma1.it (C.P.); luigi.celani@uniroma1.it (L.C.); eugenionelson.cavallari@uniroma1.it (E.N.C.); claudio.mastroianni@uniroma1.it (C.M.M.); gabriella.dettorre@uniroma1.it (G.d.); giancarlo.ceccarelli@uniroma1.it (G.C.); 2 Department of Agro-Food Science and Technology, University of Bologna, Viale Fanin 46, 40127 Bologna, Italy; l.laghi@unibo.it; 3 Department of Neurology Sapienza, University of Rome, Piazzale Aldo Moro 5, 00185 Rome, Italy; mariangela.fratino@uniroma1.it; 4 Laboratory of Virology, Department of Molecular Medicine, Affiliated to Istituto Pasteur Italia-Cenci Bolognetti Foundation, Sapienza, University of Rome, 00185 Rome, Italy; carolina.scagnolari@uniroma1.it (C.S.); federica.frasca@uniroma1.it (F.F.); guido.antonelli@uniroma1.it (G.A.); 5 Microbiology and Virology Unit, Hospital “Policlinico Umberto I”, Sapienza, University of Rome, 00185 Rome, Italy

**Keywords:** HIV, probiotics, phenylalanine, tyrosine, metabolism, inflammation, neurocognitive impairment

## Abstract

To investigate the effects of oral bacteriotherapy on intestinal phenylalanine and tyrosine metabolism, in this longitudinal, double-arm trial, 15 virally suppressed HIV+ individuals underwent blood and fecal sample collection at baseline and after 6 months of oral bacteriotherapy. A baseline fecal sample was collected from 15 healthy individuals and served as control group for the baseline levels of fecal phenylalanine and tyrosine. CD4 and CD8 immune activation (CD38^+^) was evaluated by flow cytometry. Amino acid evaluation on fecal samples was conducted by Proton Nuclear Magnetic Resonance. Results showed that HIV+ participants displayed higher baseline phenylalanine/tyrosine ratio values than healthy volunteers. A significand reduction in phenylalanine/tyrosine ratio and peripheral CD4^+^ CD38^+^ activation was observed at the end of oral bacteriotherapy. In conclusion, probiotics beneficially affect the immune activation of HIV+ individuals. Therefore, the restoration of intestinal amino acid metabolism could represent the mechanisms through which probiotics exert these desirable effects.

## 1. Introduction

Despite the widespread use of antiretroviral drugs and the subsequent achievement of viral suppression in most infected individuals, the HIV population is still facing a high prevalence of non-AIDS defining conditions related to the high levels of immune activation [1]. Growing evidences support the role of gut resident bacteria in the pathogenesis of the persistence of immune activation [2,3] and in the onset of neurologic disorders like major depression and autism, as well as in the regulation of mood and behaviour (the microbiota–gut–brain axis) [4,5,6]. Several studies performed in mice showed that a depressive phenotype can be transferred with fecal transplantation [7], confirming the profound importance of gut microbes in the regulation of brain activity. The imbalance of gut bacterial species, usually referred as dysbiosis, plays an important role in the onset of such alterations through the interference with amino acid absorption and metabolism and through the modification of the synthesis of microbiota-derived bioactive products [8]. Gut dysbiosis represents one of the hallmarks of HIV infection, even in patients on antiretroviral treatment and with undetectable levels of HIV viraemia [2]. The metabolism of aromatic amino acids such as tryptophan (precursor of serotonine), phenylalanine (Phe) and tyrosine (Tyr, precursors of catecholamines) is disrupted in HIV-1 infected individuals with subsequent implications in the pathogenesis of neurocognitive alterations in this population [9]; moreover, the modification of tryptophan catabolism observed in this population have been linked to cognitive impairment and depression, as a consequence of a reduced synthesis of serotonin and an accumulation of neurotoxic metabolites [10,11,12,13]. Phenylalanine and tyrosine represent precursors in the pathway of the synthesis of catecholamines, thus alterations in their metabolism and a diminished conversion of Phe to Tyr by the enzyme phenylalanine-hydroxylase (PAH), could lead to a reduced cognitive performance [8,14]; by the way the metabolism of these two amino acids in the setting of HIV infection has not been extensively investigated as that of tryptophan. Alterations of phenylalanine/tyrosine ratio have been observed in different inflammatory conditions such as sepsis, cancer and in the elderly, as well as in HIV infection [14]. In HIV positive individuals, an increased phenylalanine/tyrosine ratio has been recognized as a marker of immune activation and as a surrogate indicator of a reduced activity of tetrahydrobiopterin, a fundamental co-factor in the pathway of the synthesis of dopamine and the other catecholamines [15]. However, more data are necessary before firm conclusions can be drawn. Supplementation of antiretroviral (ARV) therapy with oral probiotics showed the ability to modulate gut microbial species, restoring gut mucosal immunity and thus potentially decreasing microbial translocation and immune activation [16,17,18]. Furthermore, probiotics showed the ability to improve neurocognitive function and reduce immune activation in this population [19,20]. However, it remains unclear whether probiotics supplementation can affect the metabolism of aromatic amino acids, as Phe and Tyr, in HIV-1 infected patients. Hence, given the impact of gut microbiota activity on the metabolism of aromatic amino acids involved in the regulation of cognitive function [7,8,9,10,11,12,13], and considering that neurocognitive decline can be improved with probiotics intake in HIV+ patients [19,20], we hypothesized that gut microbes composition might modulate neuroinflammation and neurocognitive functions through the effect on the balance of Phe and Tyr during HIV-1 infection. With the present study we aimed to evaluate, for the first time to our knowledge, the effect of supplementation with oral probiotics on phenylalanine and tyrosine metabolism (primary outcome) as well as its effect on neurocognitive function (secondary outcome) of HIV-1 infected individuals.

## 2. Results

### 2.1. Study Population

Overall, 15 subjects were enrolled in the study (3 not meeting inclusion criteria, 6 declined to participate). All subjects in the study were adult Caucasian males (Table 1).

The median age of HIV+ participants was 42 (range: 24–56) years old. Median time from HIV diagnosis was 12 (range: 3–28) years, while median duration of ARV therapy was 8 (range: 1–17) years; all subjects were on a stable ARV regimen since at least 6 months prior to enrollment. The median T CD4 nadir count was 247 (range: 25–560) cell/μL, while the T CD4 count at enrollment was 736 (range: 493–1315) cell/μL. All HIV+ patients had no co-infections at enrollment. Individuals in the healthy control (HC) group were age and gender matched to the HIV+ participants.

### 2.2. Effects of Probiotics on Peripheral Immune Activation

HIV+ participants underwent blood sampling at T0 and T6 to evaluate peripheral immune activation defined as the proportion of CD4^+^ and CD8^+^ T cells expressing CD38. At T6 we observed a significant decrease of CD4^+^ CD38^+^ T cells (median values: T0: 7.65% vs. T6: 4.11%, *p* = 0.012) and a trend toward the reduction of CD8^+^ CD38^+^ T cells (median values: T0: 3.70% vs. T6: 1.46%, *p* = 0.075).

### 2.3. Effects of Probiotics on Phenylalanine and Tyrosine Metabolism

HIV+ participants provided fecal samples at T0 and T6 (after 6 months of supplementation with oral probiotics).

**Figure 1:**
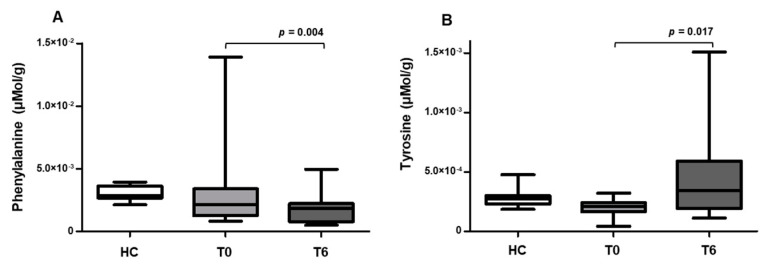
Phenylalanine and tyrosine fecal concentrations in HC and HIV+ patients before T0 and after T6 oral probiotics supplementations. (**A**) Phenylalanine fecal concentration in HC and HIV+ subjects measured before T0 and after T6 supplementation with oral probiotics. (**B**) Tyrosine fecal concentration in HC and HIV+ subjects before T0 and after T6 supplementation with oral probiotics. The line indicates median population value. Data were analyzed using the Mann–Whitney test for unpaired samples and Wilcoxon test for paired samples.

**Figure 2:**
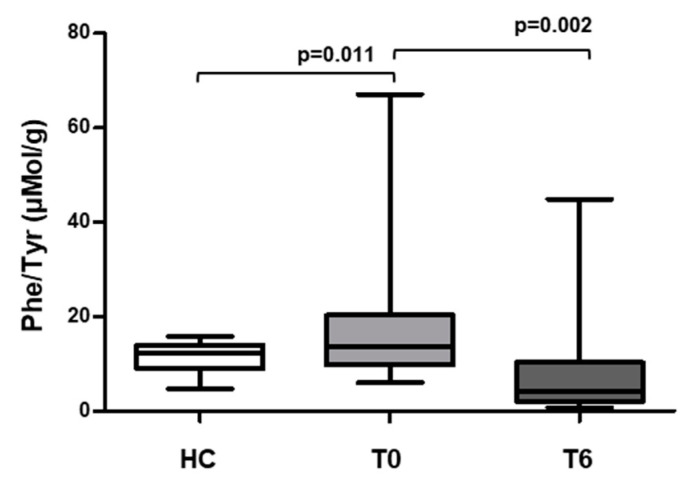
Phenylalanine/tyrosine ratio (Phe/Tyr) in HC and HIV+ subjects measured before T0 and after T6 supplementation with oral probiotics. The line indicates median population value. Data were analyzed using the Mann–Whitney test for unpaired samples and Wilcoxon test for paired samples.

The comparison of fecal phenylalanine and tyrosine concentration between HC and HIV+ patients at T0 and T6 are shown in Figure 1. Instead, the comparison of phenylalanine/tyrosine ratio (Phe/Tyr) between HC and HIV+ patients at T0 and T6 after supplementation with oral probiotics are shown in Figure 2.

At baseline HIV+ patients showed a higher Phe/Tyr ratio than healthy controls [HIV+ T0: 13.6 (6–67.1) μmol/g vs. HC: 12.2 (4.67–15.8) μmol/g; *p* = 0.011]; Figure 2) while Phe [HIV+ T0: 2.1 × 10^−3^ (0.4 × 10^−3^–2.2 × 10^−3^) μmol/g vs. HC: 2.8 × 10^−3^ (2.1 × 10^−3^–3.9 × 10^−3^) μmol/g] and Tyr levels [HIV+ T0: 2.0 × 10^−3^ (4.23 × 10^−5^–3.2 × 10^−4^) μmol/g vs. HC: 2.7 × 10^−4^ (1.8 × 10^−4^–4.7 × 10^−4^) μmol/g] were not different between the two groups analyzed (*p* > 0.05, Figure 1). After probiotics supplementation, both Phe levels [HIV+ T6: 1.85 × 10^−3^ (0.8 × 10–4.9 × 10^−3^) μmol/g] and Phe/Tyr ratio [HIV+ T6: 4.23 (0.76–44.9) μmol/g] decreased in HIV−1 infected patients (Phe: T0 vs. T6 *p* = 0.004; Figure 1; Phe/Tyr ratio: *p* = 0.002; Figure 2). By contrast, Tyr levels were higher at T6 compared to those at T0 (HIV+ T6: 3.4 × 10^−4^ (1.1 × 10^−4^–1.5 × 10^−3^ μmol/g, *p* = 0.017; Figure 1).

**Figure 3:**
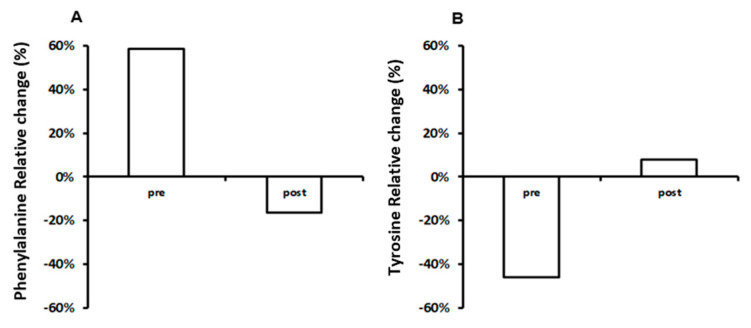
(**A**) Mean relative differences of HIV negative individuals from normal level of phenylalanine. The phenylalanine concentration has been measured in the pre (T0) and post (T6) treatment of probiotics. (**B**) Relative differences in percentage of HIV negative subjects from normal values of tyrosine. The tyrosine has been measured in the pre (T0) and post-treatment (T6). Average (grey: untreated; white: treated).

**Figure 4:**
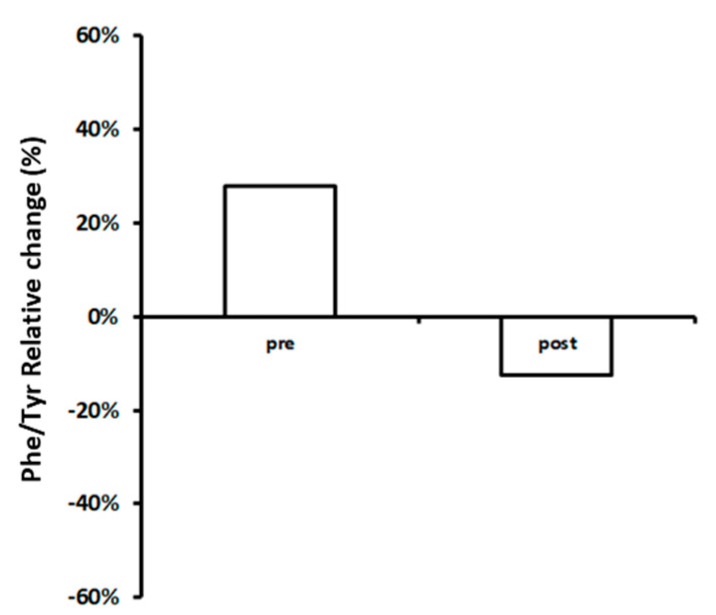
Relative differences of HIV negative individuals from normal level of phenylalanine/tyrosine ratio (Phe/Tyr) calculated before and after supplementation of probiotics. Average (grey: untreated; white: treated).

As an internal control, cut-off values based on uninfected populations were calculated to define the normal level for phenylalanine 0.8 (0.1–3.9) μmol/g, tyrosine 0.4 (0.1–0.7) μmol/g and phenylalanine/tyrosine ratio 7.2 (2.6–18.5). When we compared amino acids concentration at T0 and T6 between HIV+ subjects and normal level, we observed that HIV+ individuals showed significantly relative differences of concentrations [Phe: Pre, 60% (57–63%) vs. Post, −18% (−17.1–−18.9%); Tyr: Pre, −45% (−42.75–−47.25%) vs. Post, 10% (9.5–10.5%); Phe/Tyr: Pre, 30% (28.5–31.5%) vs. Post, 15% (14.25–15.75%)] (Figure 3 and Figure 4).

A multivariate analysis confirmed the beneficial effect of oral probiotics on phenylalanine reduction, tyrosine increase and Phe/Tyr decrease (*p* < 0.001, F 31.391).

### 2.4. Effects of Probiotics on Neuroinflammation and Cognitive Function

Cerebrospinal fluid (CSF) neopterin level and neurocognitive performance in HIV+ patients were evaluated at baseline and after six months of probiotic intake. Baseline CSF neopterin concentration was found to be above the normal threshold (9–20 nmol/L) [21] in all investigated HIV-1 positive subjects (mean value: 42.26 ± 6.78 nmol/L). Neopterin concentration in CSF was significantly reduced after supplementation with oral probiotics (mean value: 29.29 ± 13.8 nmol/L; T0 vs. T6, *p* = 0.002).

Supplementation with oral probiotics exerted a positive effect on neurocognitive function of HIV-1 infected participants (Table 2). At baseline, all HIV positive subjects showed impaired results in at least one neurocognitive test and most of them showed impairment in two different cognitive domains. At T6, all participants showed a normal neurocognitive performance with significant improvements observed in several of the performed tests (Table 2). 

### 2.5. Measure of Adherence to Probiotics Intake and Safety of the Intervention

Adherence to probiotic prescription was evaluated and confirmed with the observation of an increased fecal concentration of Bifidobacteria spp. after 2 months (T2) [T0: 7.70 (7.52–7.91) Bifidobacteria/g of feces (log10) vs. T2 8.06 (7.71–8.14) Bifidobacteria/g of feces (log10); *p* = 0.021) and 6 months of probiotics intake [T6: 8.0 (7.55–8.35) Bifidobacteria/g of feces (log10); T0 vs. T6, *p* = 0.028) in each participant. No side effects were reported during the period of probiotics intake.

## 3. Discussion

An increased risk of developing neurocognitive disorder is a common outcome related to HIV-1 infection [4,5,6,7] and a growing body of evidence supports the pivotal role played by gut microbiota in the modulation of cognitive function [22]. This influence is exerted through several mechanisms that include the synthesis of short-chain fatty acids and other bioactive metabolites by intestinal bacteria as well as the modulation of peripheral and central activity of neurotransmitters [4,5,6,7]. Phe and Tyr, that represent precursors for the synthesis of dopamine, also seem to play a critical role in the balance of neurocognitive function, as highlighted by peculiar conditions, in which PAH function appears to be impaired [23]. Accordingly, with the present study, we aimed to evaluate the fecal concentrations of phenylalanine and tyrosine and the Phe/Tyr in a population of HIV-1 infected individuals with subclinical cognitive impairment; since HIV-1 infection holds a significant impact on gut microbial species metabolism [24], we therefore evaluated the impact of long term oral probiotics intake on phenylalanine and tyrosine metabolism and the effect of probiotics on patients’ cognitive performance. To do so, we evaluated the fecal concentrations of the two amino acids in a group of HIV-1 infected participants before and after probiotic supplementation; fecal amino acid concentrations were also evaluated in a control group of age and gender matched HIV negative individuals. Subclinical neurocognitive impairment was investigated in HIV-1 infected participants with a complex neurocognitive test battery developed and administered by a neuropsychologist and with the evaluation of CSF neopterin concentration.

At baseline, we observed higher fecal Phe/Tyr and similar fecal Phe and Tyr concentrations in HIV positive participants than in healthy controls and this was not surprising, given the fact that in the setting of HIV infection Phe/Tyr is correlated to markers of systemic immune activation [14]. In this context, amino acid status of HIV-1 infected patients significantly differs from that of healthy controls and this could be explained by differences in intestinal absorption, amino acid utilization and requirement and inflammatory biomarkers [25]. Moreover, in HIV positive subjects as well as in negative individuals, phenylalanine and tyrosine represent precursors involved in the synthesis of catecholamines, such as dopamine; Phe/Tyr is usually considered a marker of the activity of tetrahydrobiopterin, the enzyme that catalyzes the first step of the pathway of catecholamines synthesis (the transformation of phenylalanine into tyrosine), therefore an elevated Phe/Tyr has been associated with a reduced level of dopamine [14] and subsequently with imbalanced cognitive abilities. Compared to total amino acids, the impairment in the enzymatic conversion of Phe is strongly related to clinical symptoms like depressive mood [26,27,28] and the worsening of life quality [29,30,31]_,_ especially in HIV-1 infected patients, due to the association between the Phe metabolism and HIV-1 pathology [18].

Accordingly, the increased Phe/Tyr observed in HIV+ participants at baseline is consistent with the subclinical cognitive alteration observed in the same group of patients and with the neuroinflammation status at CNS level, assessed by CSF neopterin measurement, which results deeply altered compared to the normal range observed in healthy adult individuals [21]. In this regard, Zangerle et al. observed a strong association between Phe/Tyr ratio and Phe and Tyr concentrations and neopterin in urine and plasma of HIV-1 infected patients that could be explained by the ability of pro-inflammatory stimuli like Interferon-γ (IFN-γ) to induce the release of neopterin, by dendritic cells and human monocyte derived macrophages [14,32]. Moreover, increased neopterin levels might in turn promote the production of large quantities of reactive oxygen species (ROS) [33]. During this condition of high oxidative stress, tetrahydrobiopterin (BH4), the natural cofactor of PAH, is destroyed, so that proper function of PAH could be altered [34].

Notably, probiotics are known to promote the restoration of gut mucosal integrity of HIV-1 infected individuals, favoring the reduction of the translocation of microbial derived products in the blood and thus exerting anti-inflammatory effects at the systemic level and in the CNS [16,17], with positive outcomes in terms of cognitive function. In our study population, oral supplementation with probiotics exerted a favorable action promoting a decrease in fecal phenylalanine and an increase in fecal tyrosine concentrations with a subsequent improvement of fecal Phe/Tyr. Of note, after probiotics supplementation HIV positive subjects showed a lower Phe/Tyr than healthy controls. Thus, as combined antiretroviral therapy (cART) leads to a decrease on HIV-1 replication, the related reduction of neuroinflammation observed in our HIV-1 infected patients could be coherent not only with neopterin concentration but also with ROS decrease and, consequently, with the reduction in oxidative stress. The latter condition could improve the activity of BH4 and boost PAH function [14,15]. One of the consequences of the above proposed mechanisms could be the increase of Tyr concentration, which is a primary intermediate of catecholamines neurotransmitters synthesis [14,15]. However, this conclusion could be limited by the fact that, in this study, any measurement of PAH or neurotransmitters levels was not performed. However, these modifications of the intestinal amino acid metabolism in HIV+ patients can partly be attributed to the modulation of the metabolic activity of the gut resident bacteria and partly to the reduction of the systemic and central immune activation demonstrated by the observation of a lower proportion of circulating activated CD4 and CD8 T cells and of a lower neopterin concentration in CSF. Interestingly, metabolic pathways of intestinal microbiota significantly have been shown to be correlate with marker of T cell activation as CD38, bacterial translocation (BPI, sCD14), LPS biosynthesis and inflammation [35]. Anyway, independently from which was the predominant mechanism, from the phenotype point of view at the end of probiotics supplementation HIV-1 infected subjects showed an amelioration of neurocognitive performance. Moreover, given the strong connection between the CNS function and the intestinal microenvironment, the analysis performed in this study aimed to obtain more insights into the gut–brain axis and to understand if and how probiotics could impact on this complex bidirectional crosstalk and act directly on T cell immune activation [36,37].

## 4. Materials and Methods

### 4.1. Study Design, Study Population and Ethical Statement

This longitudinal, double-arm study included HIV+ subjects on stable and effective cART enrolled at the HIV Outpatient Clinic of the Department of Infectious Diseases of Sapienza, University of Rome and healthy volunteers.

Out of 24 HIV+ patients screened, 15 HIV+ subjects responded to the eligibility characteristics and were enrolled in the study (3 not meeting inclusion criteria, 6 declined to participate). All subjects in the study were adult Caucasian males. As an internal control, phenylalanine and tyrosine concentrations were measured in 15 age and gender matched healthy uninfected subjects, following the same nutritional habits as the HIV+ group and with no neurological and behavioral alterations

The study was approved by the internal committee of the Public Health and Infectious Diseases Department and by the Ethics Committee of Sapienza, University of Rome (Ref. 2970). Prior to the enrolment, all HIV+ and healthy participants gave their written informed consent to be included in the study.

### 4.2. Eligibility Criteria

Inclusion criteria for the enrollment in the study were: age >18 years old, a stable (at least 6 months prior to inclusion) and effective (plasma HIV RNA <37 copies/mL) HAART regimen. Exclusion criteria were: education below primary school, Mini Mental State Examination <26, any impairment in daily living activities as defined in the Instrumental Activities of Daily Living scale, previous history or actual diagnosis of any neurologic or psychiatric condition, positive PCR on CSF for any of the following pathogens: Citomegalovirus (CMV), Epstein-Barr virus (EBV), Herpes simplex virus 1 (HSV1), Herpes simplex virus 2 (HSV2), Varicella-zoster virus (VZV), Human herpes virus 8 (HHV8), BK virus (BKV), John Cunningham virus (JCV).

### 4.3. Study Timeline and Investigational Compound

All HIV+ participants underwent blood, fecal and CSF sampling and neurocognitive evaluation at baseline (T0) and after six months (T6) of supplementation with a commercially available multistrain probiotic formulation (trade name: Vivomixx^®^ in EU and Visbiome^®^ in USA. Composition: *Streptococcus thermophilus* DSM24731, *Bifidobacterium breve* DSM24732, *Bifidobacterium longum* DSM24736, *Bifidobacterium infantis* DSM24737, *Lactobacillus acidophilus* DSM24735, *Lactobacillus plantarum* DSM24730, *Lactobacillus paracasei* DSM24733, *Lactobacillus delbrueckii ssp. bulgaricus* DSM24734;). HIV+ patients also underwent fecal sampling after two months of probiotic supplementation (T2) to evaluate the adherence to probiotics intake and the intervention safety. The control group underwent fecal sampling at T0.

During the study period, participants were told not to change their usual dietary habits with particular regard to phenylalanine and tyrosine rich foods.

### 4.4. Peripheral Immune Activation

PBMC were collected and aliquoted in 1 × 10^6^ cells/mL RPMI medium plus 10% Fetal Bovine Serum and then washed by centrifugation. The following anti-human monoclonal antibodies were added: CD3-PerCP, CD4^+^-APC-Vio770, CD8^+^-FITC, CD4^+^ 5RO-PEVio770, CD27-VioBlue, CD38-APC, (Miltenyi Biotec, Bergisch Gladbach, Germany). Samples were acquired by Miltenyi Biotec flow cytometer-MACSQuant Analyzer (8 fluorescence channels, 3 lasers). Gating and data analysis were performed using MACSQuantify software 2.5 (Miltenyi Biotec, Bergisch Gladbach, Germany).

### 4.5. Fecal Metabolome

**Figure 5:**
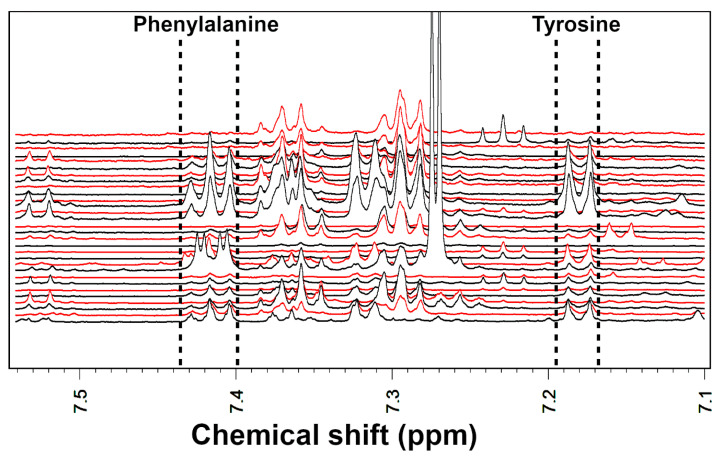
Portions of the NMR spectra employed for the quantification of phenylalanine and tyrosine. Samples collected at T0 and T6 are represented with black and red colours, respectively.

For Nuclear Magnetic Resonance (NMR) analysis, 80 mg of frozen stool samples were mixed with 1 mL of deionized water by vortex mixing them for 5 min, followed by centrifugation for 15 min at 18,000 g and 4 °C. 100 μL of a D_2_O solution of 3-(trimethylsilyl)-propionic-2,2,3,3-d4 acid sodium salt, 10 mM, set at pH 7.00 with 1-M phosphate buffer were added to about 700 mL of supernatant. Before analysis, the samples were again centrifuged. Proton NMR (^1^H-NMR) spectra were recorded at 298 K with an AVANCE III spectrometer (Bruker, Milan, Italy) operating at a frequency of 600.13 MHz. The Hydrogen Deuterium Oxide residual signal was suppressed by presaturation, whereas broad signals from slowly tumbling molecules were removed by including a Carr–Purcell–Meiboom–Gill filter [38] to a free induction decay sequence. The filter was made up by a train of 400 echoes separated by 800 μs, for a total time of 328 ms. Each spectrum was acquired by summing up 256 transients using 32 K data points over a 7211.54-Hz spectra (for an acquisition time of 2.27 s). The recycle delay was set to 8 s, considering the longitudinal relaxation time of the protons under investigation. A representative ^1^H-NMR spectrum for phenylalanine and tyrosine quantification was reported in Figure 5. Spectra were adjusted for phase and baseline in Topspin ver. 3.5 (Bruker, Milan, Italy). Any other further processing was performed in R computational language (www.r-project.org) as detailed by Foschi et al. [39]. In detail, molecules’ quantification was performed in the first sample acquired by employing the added Trimethylsilylpropanoic acid (TSP) as an internal standard. In order to compensate for differences in solids content, any other sample was then normalized to such sample by means of normalized absolute quantification [40]. Integration of the signals was performed for each molecule by means of rectangular integration.

### 4.6. Bacterial DNA Isolation from Fecal Samples

Bacterial DNA was evaluated on fecal samples in order to assess patients’ adherence to probiotics intake and to evaluate the efficacy of the product to modify intestinal microbiota composition. For this purpose, QIAamp DNA Stool Mini Kit (Qiagen, Hilden, Germany) was used according to the manufacturer’s instructions: 200 mg of frozen samples were suspended in 1.4 mL of ASL lysis buffer from the stool kit, added with glass beads (150–212) μm, (Sigma-Aldrich, St. Louis, MO, USA), and homogenized. The suspension was incubated at 95 °C for 5 min, DNA was purified and eluted in 200 μL of AE buffer and the samples obtained were stored at −20 °C. Finally, bacterial DNA from faecal samples was quantified by a real-time PCR, performed to evaluate Bifidobacteria levels. Briefly, PCR amplification and detection were performed on optical-grade 96-well plates, using the Applied Biosystems 7500 Real-Time PCR instrument (Applied Biosystems, Inc., Norwalk, CT, USA). The reaction mixture was composed of SensiMix SYBR Low-ROX (BIOLINE, Taunton, MA, USA), 500 nM primers for Bifidobacterium genus, and 2.5 μL of template DNA (final volume = 25 μL). After amplification, a melting curve analysis was made to distinguish target amplicons from aspecific non-target PCR products. Standard curves were made by using 10-fold dilutions of DNA, extracted from Bifidobacterium breve. All samples were analysed in duplicate in two independent real-time PCR assays.

### 4.7. ELISA Assay for Evaluation of CSF Neopterin Levels

CSF was collected by lumbar puncture and cell-free centrifuged supernatant samples were stored at −80 °C. CSF neopterin levels were determined by a commercially available solid phase enzyme-linked immunosorbent assay (ELISA), based on the basic principle of a competitive ELISA (IBL International GmbH, Hamburg, Germany), as previously described [19].

### 4.8. Neurocognitive Evaluation

A trained neuropsychologist carried out several neuropsychological tests to all HIV+ patients in order to explore ability in the following areas: verbal, language, attention, learning memory, working memory, abstraction, executive functions, processing speed of information, sensory-perceptual and motor. The test battery included: Rey-Osterrieth Complex Figure Test (ROCF) to evaluate participants’ recognition and recall skills for non-verbal contents, Rey Auditory Verbal Learning Test (RAVLT) to evaluate short term auditory-verbal memory, rate of learning and retention of information, Test of Weights and Measures Estimation (STEP) to evaluate abstraction skills, Visual Search Test (Attention Matrices Test) to evaluate attention skills, Verbal Fluency test (FAB) to evaluate executive functions and the ability to switch between different tasks, Test of Phonological and Semantic Verbal Fluency (respectively PVF and SVF) to evaluate phonological and semantic supplies and the ability to access them, Raven’s Standard Progressive Matrices (SPM) to evaluate abstract reasoning and problem solving capabilities, Digit Span test to evaluate short term memory and executive functions, Corsi Block Tapping Test (CBTT) to evaluate short term spatial memory and executive functions, Aachener Aphasia Test (AAT) to evaluate the presence of aphasia among study participants, Trail Making Test A and B (TMT A and TMT B) to evaluate visual-spatial attention and motor skills.

### 4.9. Statistical Analysis

Data are presented as Mean and standard deviation (SD) or median and range (minimum-maximum). Paired sample *t*-test or Wilcoxon signed-rank test were applied to evaluate paired samples between T0 and T6. A Kolmogorov–Smirnov test was applied to verify the normal distribution of values of the considered variables and, based on the evidence obtained, independent sample t-test was subsequently applied to analyse variables that showed a normal distribution, while the Wilcoxon signed-rank test test was applied in the case that the variables did not show a normal distribution. A multivariate analysis was the conducted to evaluate the impact of probiotic intake on the observed modifications of amino acids concentration to assess significance, an F test that accounted for design effects has been used.

## 5. Conclusions

The results from our study are consistent with previously reported data that showed a beneficial effect of probiotics on systemic immune activation, CNS inflammation and neurocognitive function. Despite the small sample size and the lack of the neurotransmitters concentration measurement, our study highlights the beneficial effect of this compound on cognitive performance in virologically suppressed HIV-1 infected individuals; we speculated that this could be at least partially explained by the modulation of tetrahydrobiopterin activity and the restoration of the dopamine synthesis pathway. Accordingly, probiotic supplementation deeply impacts on the metabolism of the essential amino acid Phe, which is severely altered during HIV-1 infection [9], improving its conversion to Tyr. Nevertheless, our data further support previous evidence about the advantageous effect of adjunctive therapies on T cell immune activation and gut epithelial integrity recovery in HIV-1 infected patients [41,42]. Further investigations are needed to confirm our preliminary data and to investigate probiotics role in the setting of symptomatic neurocognitive impairment in order to introduce this therapeutic strategy to everyday clinical practice and improve the complex management of this population.

## Figures and Tables

**Table 1:** Characteristics of HIV+ subjects enrolled in the study.

Characteristics	HIV+ Patients ^a^	Healthy Controls
N of subjects	15	15
Males	15	15
Age	42 (24–56)	41 (25–57)
Years from diagnosis	12.4 (3–28)	NA
Years on ARV treatment	8 (1–17)	NA
T CD4 nadir	247 (25–560) cell/μL	NA
T CD4 at enrollment	736 (493–1315) cell/μL	NA
Therapy class (number)	PI (6/15) NRTI (11/15) NNRTI (4/15) NtRTI (5/15) INSTI (4/15)	NA

**^a^** Data were expressed as median values; range is reported between brackets). Abbreviations: ARV: antiretroviral; CD4: cluster of differentiation 4, PI: Protease Inhibitor; NRTI: Nucleoside Reverse transcriptase inhibitor; NNRTI: Non-nucleoside reverse-transcriptase inhibitor; NtRTI: Nucleotide reverse transcriptase inhibitor; INSTI: Integrase Strand Transfer Inhibitor.

**Table 2:** Neurocognitive tests performance of HIV+ participants.

Neurocognitive Tests	T0 ^a^	T6 ^a^	Statistical Significance
Rey–Osterrieth Complex Figure (immediate recall)	16.4	22.1	**0.002**
Rey–Osterrieth Complex Figure (delayed recall)	16.2	23.3	**0.002**
Rey Auditory Verbal Learning Test (immediate recall)	43.0	52.7	0.460
Rey Auditory Verbal Learning Test (delayed recall)	8.6	11.9	0.054
Rey Auditory Verbal Learning Test (recognition)	94.5	98.9	0.099
Verbal Fluency	14.8	15.6	0.364
Phonological Verbal Fluency	30.1	42.6	**0.035**
Semantic Verbal Fluency	44.5	47.7	**0.034**
Visual Search Test (attentive matrices)	50.2	47.9	0.079
Test of Weights and Measures Estimation (time)	17.5	23.0	0.400
Test of Weights and Measures Estimation (weight)	17.5	22.0	**0.034**
Test of Weights and Measures Estimation (total)	37.1	44.7	0.731
Raven’s Standard Progressive Matrices	27.0	30.6	0.202
Verbal Span (forward)	4.6	5.3	0.285
Verbal Span (backward)	4.4	4.6	1.000
Corsi Block Tapping Test (forward)	4.6	5.2	0.117
Corsi Block Tapping Test (backward)	3.7	4.12	0.351
Aachener Aphasia Test	9.0	9.0	1.000
Trail Making Test A	54.3	46.0	0.120
Trail Making Test B	127.1	108.9	0.413

^a^ Data were expressed as mean values. Significant differences (*p* < 0.05) were highlighted in bold.
